# Evaluation of the Intestinal Transport of a Phenylethanoid Glycoside-Rich Extract from *Cistanche deserticola* across the Caco-2 Cell Monolayer Model

**DOI:** 10.1371/journal.pone.0116490

**Published:** 2015-02-03

**Authors:** Yuan Gao, Chuanjie Zong, Fen Liu, Lei Fang, Runlan Cai, Yue Shi, Xi Chen, Yun Qi

**Affiliations:** 1 Department of Research Center for Pharmacology and Toxicology, Institute of Medicinal Plant Development, Chinese Academy of Medical Sciences & Peking Union Medical College, Beijing, P.R. China; 2 Heilongjiang University of Chinese Medicine, Harbin, Heilongjiang, P.R. China; Cincinnati Children’s Hospital Medical Center, University of Cincinnati College of Medicine, UNITED STATES

## Abstract

Phenylethanoid glycosides (PhGs), a class of polyphenolic compounds, are considered one of major bioactive constituents of *Cistanche deserticola* Y.C. Ma (CD), whose extract is orally used in traditional Chinese medicine. Although previous pharmacological studies have reported that PhGs exert many activities, their intestinal transport profiles have not been clarified. In this study, we investigated the intestinal permeability of a PhG-rich extract (PRE) from CD as an integrated system in the Caco-2 cell monolayer model using a bioassay system. The results showed that PRE is primarily transported via poorly absorbed passive diffusion down a concentration gradient without efflux, which provides the pharmacokinetic basis for the clinical application of PhGs in CD. We also determined the intestinal permeability of three major PhGs [acteoside (AC), isoacteoside (IS) and echinacoside (EC)] by HLPC. Furthermore, we developed a novel HPLC-fluorescence detection method to accurately determine the flux amount of AC and IS. As expected, the transport characteristics of the three PhGs are consistent with those of PRE, indicating that the present bioassay system is appropriate and reliable for the evaluation of the transport characteristics of active ingredient groups (AIG) in PRE. Moreover, this system may also be suitable for other plant extracts given appropriate bioactivity.

## Introduction

The Caco-2 cell line, which was derived from human colon adenocarcinomas, exhibits enterocyte-like characteristics. Under normal conditions, Caco-2 cells spontaneously differentiate from mature cells and form intact monolayers [1]. The adjacent cells adhere via tight junctions formed at the apical side of the monolayer, which can discriminate the passively and actively transported drugs across the epithelial layer [2]. Due to the morphological and biochemical similarity to normal enterocytes, Caco-2 cell monolayers serve as a well-accepted *in vitro* model for the study of the intestinal absorption potential and transport characteristics of drugs [3, 4].

In contrast to chemicals, plant extracts (PE) are mixtures whose biological activity and active constituents are often not well identified [5]. Moreover, the intestinal transport properties of PE, as opposed to the properties of its constituents, are closely related to clinical use. Flux measurements for a test sample across a Caco-2 cell monolayer commonly involve chemical methods, such as HPLC, LC/MS, etc. Although these methods are powerful tools, they are complex, time-consuming, expensive, and occasionally require sophisticated equipment. More importantly, neither a single nor a minority component can reflect PE as a whole. Thus, a novel approach independent of the determination of constituents needs to be established to identify and evaluate the transport characteristics of PE.

*Cistanche deserticola* Y.C. Ma (CD), a holoparasitic plant, is a common traditional Chinese medicine mainly used to treat kidney deficiency, body weakness and constipation, and these uses have been officially recorded in the Chinese Pharmacopoeia [6]. Phenylethanoid glycosides (PhGs), including echinacoside (EC), acteoside (AC) and isoacteoside (IS), etc., are a class of polyphenolic compounds [7]. They are considered one of major bioactive constituents of Cistanche species [8]. Pharmacological studies have shown that the bioactivity of PhGs is diverse and includes anti-oxidative [9], anti-fatigue [10], hepatoprotective [11], immunomodulatory [12], anti-inflammatory [7, 13] and neuroprotective effects [14]. However, the intestinal transport characteristics of PhGs have not been investigated. In this study, we explored the intestinal permeability of a PhG-rich extract (PRE) from CD as an integrated system and the permeability of three major PhGs (AC, IS and EC) in differentiated Caco-2 cells. Our results indicated that PRE is primarily transported via poorly absorbed passive diffusion down a concentration gradient without efflux, which provides the pharmacokinetic basis for the clinical application of PhGs in CD.

## Materials and Methods

### Materials

The human intestinal Caco-2 cell line was obtained from the American Type Culture Collection (ATCC, Rockville, MD, USA). AC, IS and EC (>98%) were purchased from Must Bio-technology Co. (Chengdu, China). Dulbecco’s modified Eagle’s medium (DMEM), fetal bovine serum (FBS) and non-essential amino acids (NEAA) were produced by Gibco BRL (Grand Island, NY, USA). 6-well Transwell^TM^ plates (insert membrane growth area 4.67 cm^2^) were obtained from Corning (Costar) Inc. (Tewksbury, MA, USA). Rat-tail collagen was obtained from Sigma-Aldrich (St. Louis, MO, USA). All reagents and chemicals for the HPLC analysis were of analytical grade.

### Preparation of PRE from CD

The air-dried CD material was powdered and extracted by percolation with 70% ethanol. The PhG-rich fraction was prepared as previously described [10] and extracted with water-saturated n-butyl alcohol. The extract liquor was concentrated and dried under reduced pressure. Macroporous resin-UV spectrophotometry [15] measured a PhG content of 78.4%. The final sample represented a 1.75% yield of raw material by dry weight. The obtained sample was stored at −20°C until further use.

### Determination of AC, IS and EC by HPLC

A Shimadzu HPLC system equipped with the LC solution software was used to assay the contents of AC, IS and EC in PRE. A reverse phase Intersil C_18_ column (4.6 mm × 250 mm, 5 μm) was used and maintained at room temperature. The mobile phases were acetonitrile and water containing 0.1% phosphoric acid (v/v) with a gradient elution (Table 1) at a flow rate of 1.0 ml/min. The UV spectrophotometer detector was set to 334 nm. To accurately determine the flux of AC and IS, we developed a novel HPLC-fluorescence detection (HPLC-FLD) method. After a structural analysis and fluorescence wavelength scanning (data not shown), we obtained the optimal fluorescence detection conditions for AC (Ex: 338 nm, Em: 448 nm) and IS (Ex: 320 nm, Em: 434 nm).

**Table 1:** Mobile phase condition of chromatographic separation.

Time (min)	Acetonitrile (%)	0.1% phosphoric acid in H_2_O (%)
0–25	15→18	85→82
25–60	18→30	82→70

The HPLC analytical method was validated using the following performance characteristics: stability, linearity, sensitivity, precision (intra- and inter-day variability) and accuracy. The analytes in the transport buffer were stored at 25°C in the dark for 24 h, and their stability was measured. The analytes were highly stable in the presence of vitamin C (0.4%) because the relative standard deviation (RSD) values in the peak areas were < 3.5%. The linear regression equation, correlation coefficient, range of linearity, LOD and LOQ for AC, IS and EC are shown in Table 2. The LODs (18–30 nM) and LOQs (60–100 nM) values illustrate that the HPLC-FLD and HPLC-UV methods are highly sensitive. The RSD values that express the precision of the method were all < 2% for intra- and inter-day variability, indicating good precision. For recovery evaluation, AC, IS and EC were added to the transport buffer to yield three (high, medium and low) concentration levels. The recovery values of the method for the analytes are summarized in Table 3. The average recoveries ranged from 90.0% to 96.4%, indicating good accuracy. In conclusion, the established HPLC methods are satisfactory with respect to linearity, sensitivity, precision and accuracy for the quantification of AC, IS and EC in transport buffer.

**Table 2:** Linearity and sensitivity data for AC, IS and EC assays by HPLC.

Analytes	Detector	Linear equation (y = mx+n)^a^	R^2^	Range (μM)	LOD (nM)	LOQ (nM)
AC	FLD	y = 27234x+1241	0.9999	0.0625–8.0	18	60
IS	FLD	y = 25765x-300.4	0.9999	0.0625–4.0	20	62.5
EC	UV	y = 21965x+98.02	1	0.1–8.0	30	100

^a^ y: peak area; x: concentration; m: slope; n: intercept

**Table 3:** Recovery for AC, IS and EC (n = 3).

Analytes	Spiking level (μM)	Recovery (%)^a^ (n = 3)			Average (%)	RSD (%)
AC	8.0	90.0	95.6	90.4	92.3	3.26
	4.0	94.0	96.6	95.0	95.2	1.15
	1.0	85.6	91.6	93.0	90.0	3.55
IS	8.0	94.8	92.0	95.5	94.1	1.62
	4.0	90.0	88.4	94.1	90.8	2.66
	1.0	99.0	93.3	95.9	96.1	2.41
EC	8.0	94.3	98.7	93.9	95.6	2.26
	4.0	95.5	96.7	96.7	96.4	0.64
	1.0	96.2	94.4	97.9	96.2	1.44

^a^ Recovery (%) = [(measured concentration for spiked sample)/(spiked concentration)] × 100

### Determination of PRE by bioassay system

The bioassay (total anti-oxidative capacity) was based on the FRAP (ferric reducing/antioxidant power) assay we previously described [16] and validated in terms of linearity and precision (intra- and inter-day variability). The linearity of the bioassay method was determined based on the calibration curves. The concentration range of linearity was 0.0625 − 40 μg/ml (y = 0.0258x+0.0968, R^2^ = 0.996). The precision of the established bioassay method was determined in terms of the intra- and inter-day variability for an analysis of PRE (10.0 μg/ml). The intra-day repeatability of the method was determined based on five consecutive determinations on the same day. The inter-day repeatability was measured based on five consecutive determinations on three different days. The RSD values for the precision of the method were 1.014% and 4.72% for intra- and inter-day variability, respectively, indicating good precision. Thus, the established bioassay method is satisfactory with respect to linearity, precision and accuracy for the quantification of PRE in transport buffer.

### Cell culture

Caco-2 cells were cultured at 37°C and 95% relative humidity in an atmosphere containing 5% CO_2_ and medium consisting of DMEM, 10% FBS, 1% NEAA, 1% L-glutamine, penicillin (100 U/ml) and streptomycin (100 μg/ml). The medium was changed every other day during cell growth and differentiation. The cells were grown in 75-cm^2^ plastic flasks and harvested every 3–5 days with 0.05% EDTA-trypsin. For the transport experiments, the cells were seeded at a density of 1×10^5^ cells/cm^2^ on Transwell inserts coated with collagen. Approximately 21 days after seeding, the monolayers were used for the transport experiments. Their integrity was determined by measuring the trans-epithelial electrical resistance (TEER) across the monolayers with an EVOM equipped with ENDOHM-SNAP (World Precision Instruments, Inc., USA). The TEER values of the Caco-2 cell monolayer needed to exceed 500 Ω∙cm^2^.

### Transport experiments

The monolayer was washed with transport buffer (P-buffer containing 10 mM HEPES, 1 mM sodium pyruvate, 10 mM glucose, 3 mM CaCl_2_ and 145 mM NaCl, pH 7.4) and then pre-incubated for 20 min at 37°C. After removing the transport buffer, fresh transport buffer containing test samples was added to the apical (AP) chamber (1.5 ml) in AP to basolateral (BL) directional studies or the BL chamber (2.5 ml) in BL to AP directional studies. For AC, IS and EC assays using HPLC, an aliquot (300 μl) was removed from each receiver chamber at different time intervals (30, 60, 90 and 120 min). The receiver chamber was replenished with the same volume of fresh preheated (37°C) transport buffer after each sampling. The collected samples were stored at −20°C for further use. For PRE determination using bioassay, only the samples at 120 min were taken. Due to the instability of PRE, AC, IS and EC, 0.4% (w/v) vitamin C was added to stabilize the samples in order to determine the AC, IS and EC contents by HPLC, and the samples for the PRE bioassay were assayed immediately after being sampled. The apparent permeability coefficient (*P*_app_ or *’P*_app_) value of each sample was calculated.

### Data analysis

The *P*_app_ values in the AP →BL or BL→AP directions of AC, IS and EC were calculated based on the following equation: *P*_app_ = (△Q/△t)/(A·C_0_), where *P*_app_ is the apparent permeability coefficient (cm/s) determined by HPLC. (△Q/△t) is the rate of appearance of the test compound (AC, IS or EC) on the receiver side (μmol/s); A is the surface area of the insert (cm^2^); C_0_ is the initial test compound concentration on the donor side (μmol/ml). Specifically, the mass unit “μg” was used instead of “μmol” when the *’P*_app_ values were calculated. The data are expressed as the means ± SD.

## Results

### Composition of AC, IS and EC in PRE from CD

**Figure 1:**
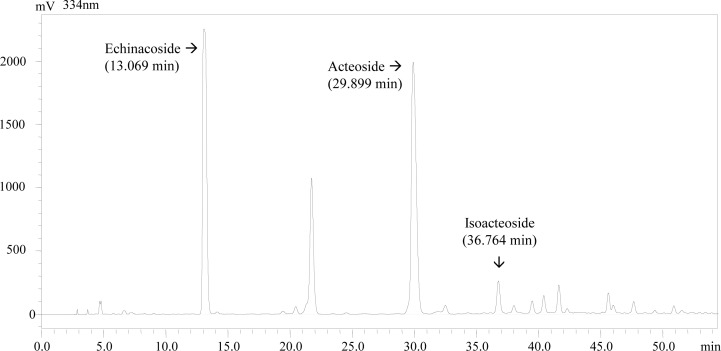
A representative chromatogram and UV spectrum of PRE at 334 nm. The mobile phases were acetonitrile and water containing 0.1% phosphoric acid (v/v) with a gradient elution at a flow rate of 1.0 ml/min.

Because AC, IS and EC are considered the major bioactive PhGs of Cistanche species [8], we analyzed their contents in PRE via HPLC-UV. The representative chromatogram is shown in Fig. 1. The identification of these constituents was based on comparing the retention times and the UV spectrum with those of authentic standards at a wavelength of 334 nm. The contents of AC, IS and EC in PRE were 26.60%, 1.84% and 32.83%, respectively. Thus, these three compounds account for 61.27% of PRE; furthermore, the above results indicate that the PhG content in PRE is 78.4%. Therefore, AC, IS and EC account for approximately 80% of PhGs in PRE.

### Total anti-oxidative capacities of PRE, AC, IS and EC

**Figure 2:**
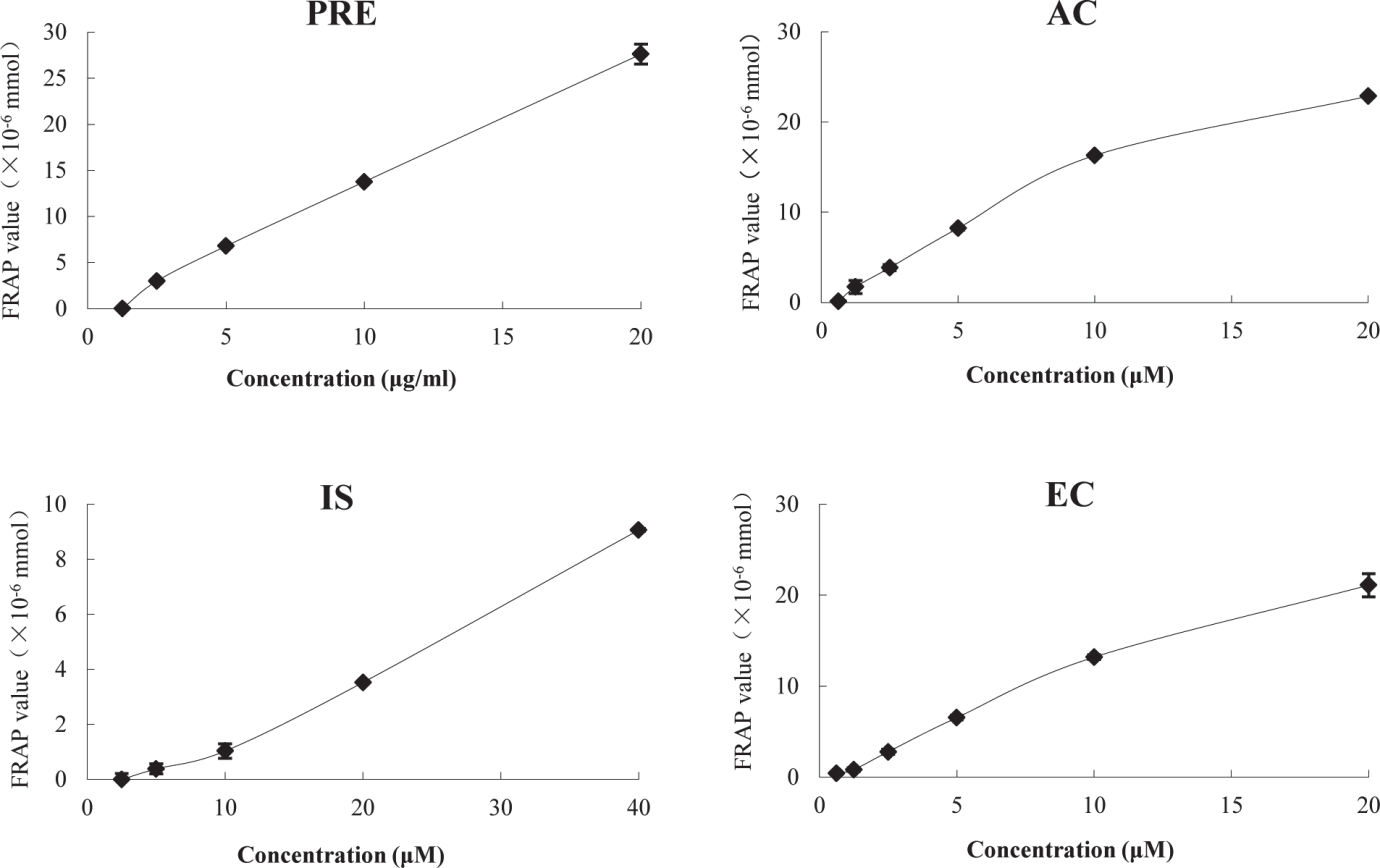
The total anti-oxidative capacities of PRE, AC, IS and EC. The data are means ± SD.

Studies of the anti-oxidative activity of PhGs from CD have been reported [9], and AC, IS and EC account for approximately 80% of PhGs in PRE. Thus, the total anti-oxidative capacities of PRE, AC, IS and EC were assayed and are expressed using the FRAP values (× 10^-6^ mmol). As shown in Fig. 2, when the FRAP value was 8 × 10^–6^ mmol, the final concentrations of PRE, AC, IS and EC were 6.20 μg/ml, 3.14 μg/ml, 22.92 μg/ml and 4.89 μg/ml, respectively, indicating that the total anti-oxidative capacity of these four samples ranked as follows: AC > EC > PRE > IS. The bioactivity of PRE is attributed to its active ingredient groups (AIG). For approximately 60% of PRE, AC and EC showed stronger total anti-oxidative activity than PRE and IS. Because IS (1.84%) constitutes a very small fraction of PRE, other components, such as the weakly anti-oxidative IS, are also clearly active in PRE. Surprisingly, the total anti-oxidative activity differed by nearly 7-fold between AC and its isomer IS, indicating that not only the number of phenolic hydroxyl groups [9] but also the position of phenolic hydroxyl groups in the molecule affected the anti-oxidative effect.

### Validation of the Caco-2 monolayers

To validate the Caco-2 cell monolayer system, the *P*_app_ values of propranolol (a well-transported marker) and Lucifer yellow (a poorly transported marker) from the AP to the BL across the Caco-2 monolayers were determined as 1.51 × 10^−5^ cm/s and 2.8 × 10^−7^ cm/s, respectively, and these values agreed with those published in previous reports [17, 18]. The alkaline phosphatase activity assay also confirmed that the Caco-2 cell monolayers were qualitatively comparable to the small intestinal epithelium [19].

### The permeability of AC, IS and EC

**Figure 3:**
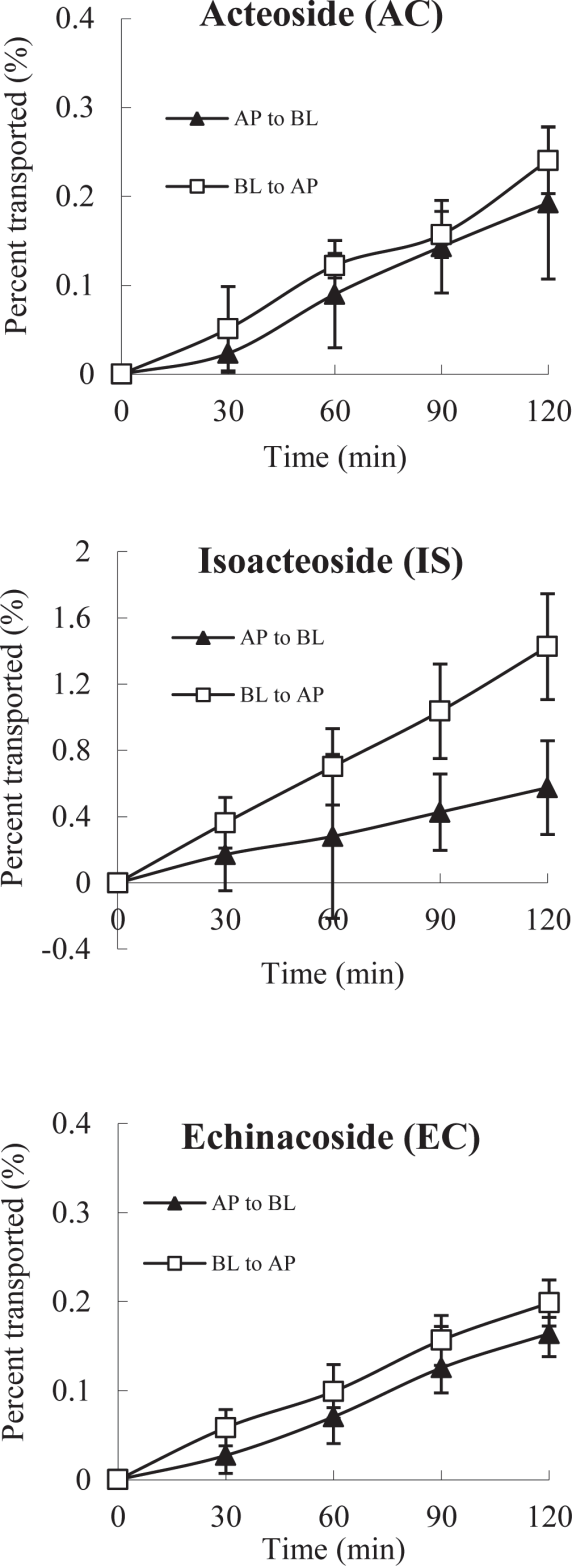
The percent of AC, IS and EC transported in Caco-2 cell monolayer as a function of time at 200 μM. The monolayer was washed with transport buffer and then pre-incubated for 20 min at 37°C. After removing the transport buffer, fresh transport buffer containing test samples was added to the AP chamber for the AP-to-BL directional studies or the BL chamber for the BL-to-AP directional studies. An aliquot sample (300 μl) was removed from each receiver chamber at different time intervals. The receiver chamber was replenished with the same volume of fresh preheated transport buffer (37°C) after each sampling. The collected samples were stored at −20°C for further use. Vitamin C [0.4% (w/v)] was added to stabilize the samples. The *P*_app_ value of each sample was calculated. All experiments were carried out in triplicate. The data are means ± SD.

**Figure 4:**
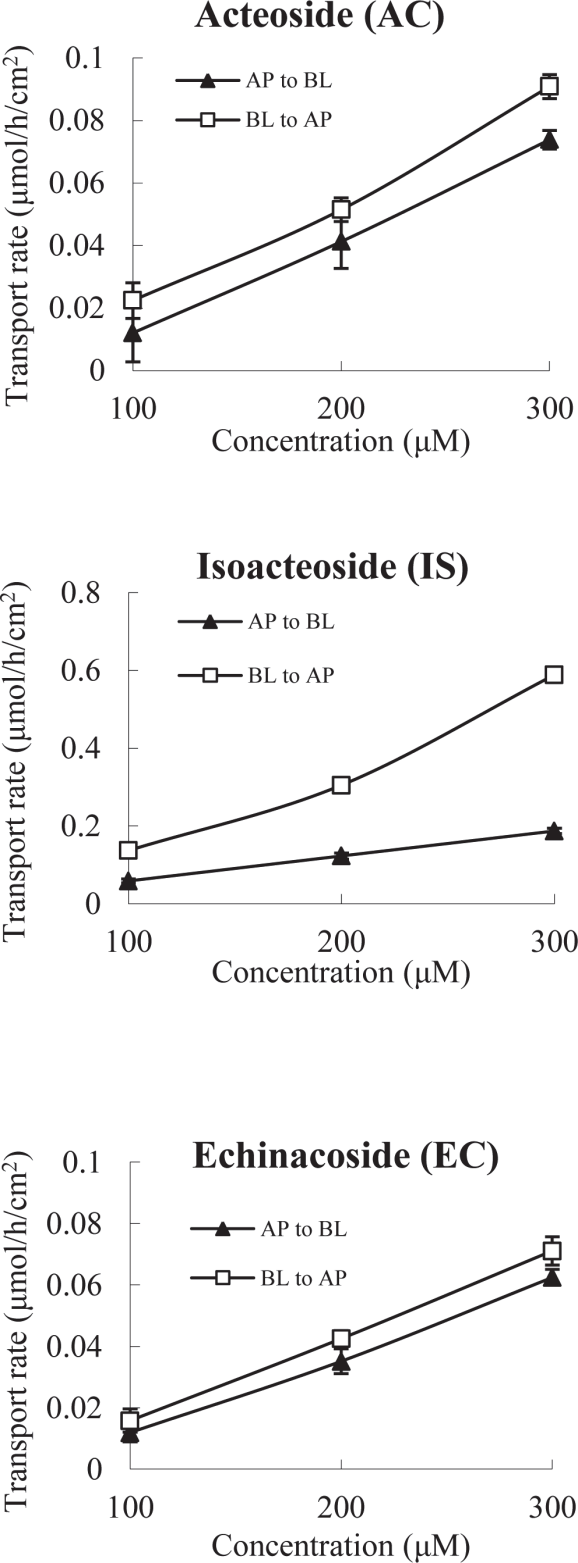
The percent of AC, IS and EC transported in the Caco-2 cell monolayer as a function of concentration at 120 min. The monolayer was washed with transport buffer and then pre-incubated for 20 min at 37°C. After removing the transport buffer, fresh transport buffer containing the test samples was added to the AP chamber for the AP-to-BL directional studies or the BL chamber for the BL-to-AP directional studies. The samples were removed from each receiver chamber at 120 min and stored at −20°C for further use. Vitamin C [0.4% (w/v)] was added to stabilize the samples. The *P*_app_ value of each sample was calculated. All experiments were carried out in triplicate. The data are means ± SD.

In general, the *P*_app_ values of well-absorbed drugs were high (> 1.0 × 10^−5^ cm/s), whereas those of poorly absorbed drugs were low (< 1.0 × 10^−6^ cm/s) [3]. As shown in Table 4, the *P*_app_ values of AC, IS and EC were nearly on the order of 10^−7^ cm/s, indicating that these compounds were poorly permeable. Furthermore, efflux or active transport were not observed because the ratios of *P*_app BL to AP_ / *P*_app AP to BL_ for AC, IS and EC were between 0.90 ~ 1.55, and the criterion of net efflux proposed by the FDA Guidance is a ratio less than 2 [20]. Based on the kinetic curves presented in Fig. 3, the bidirectional transport percentages of AC, IS and EC at 200 μM increased linearly with time. The transport rate (TR) values of the three compounds increased linearly in both directions between approximately 100 and 300 μM (Fig. 4). These results indicate that the main transport mechanism of AC, IS and EC is also passive diffusion.

**Table 4:** The bidirectional *P*_app_ values of AC, IS and EC in the Caco-2 cell monolayer model.

Compounds	*P*_app AP to BL_ (×10^−7^, cm/s)	*P*_app BL to AP_ (×10^−7^, cm/s)	*P*_app BL to AP_ / *P*_app AP to BL_
AC	1.15 ± 0.08	1.07 ± 0.03	0.93
IS	4.10 ± 0.32	6.36 ± 0.69	1.55
EC	0.98 ± 0.01	0.89 ± 0.02	0.90

### The permeability of PRE

**Figure 5:**
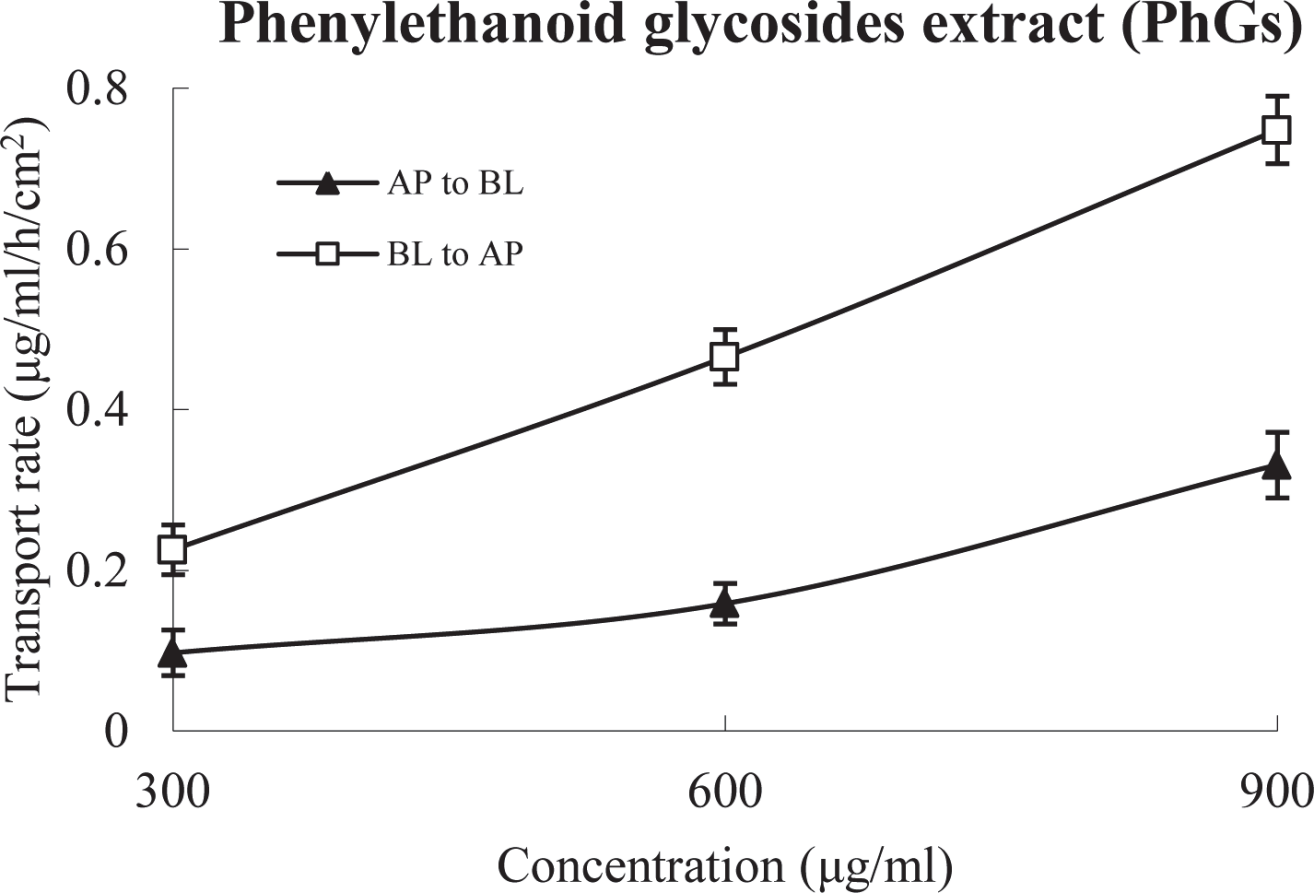
The percent of PRE transported in the Caco-2 cell monolayer as a function of concentration at 120 min. The monolayer was washed with transport buffer and then pre-incubated for 20 min at 37°C. After removing the transport buffer, fresh transport buffer containing the test samples was added to the AP chamber (1.5 ml) for the AP-to-BL directional studies or the BL chamber (2.5 ml) for the BL-to-AP directional studies. The sample was removed from the receiver chamber at 120 min and immediately assayed. The *’P*_app_ value was calculated. All experiments were carried out in triplicate. The data are means ± SD.

The above results indicate that the total anti-oxidative capacity of PRE is at least due to 61.27% of AIG from PRE. Thus, we evaluated the TR of PRE based on its concentration-effect curve of total anti-oxidative capacity and calculated the *’P*_app_ values in order to reflect the transport characteristics of PRE. The *’P*_app_ values of PRE for AP→BL and BL→AP were (2.16 ± 0.26) × 10^−7^ cm/s and (3.13 ± 0.29) × 10^−7^ cm/s, respectively, indicating that this compound was poorly permeable. Furthermore, efflux or active transport was not evident because the ratio of *’P*_app BL to AP_ / *’P*_app AP to BL_ for PRE was 1.45 [20]. Moreover, the bidirectional TR of PRE increased linearly between approximately 300 and 900 μg/ml (Fig. 5). The lack of directional preference of the results suggests that passive diffusion is the main transport mechanism of PRE.

## Discussion

Compared with highly purified drug products, PE are generally mixtures that consist of hundreds of constituents with widely different physiochemical properties. Therefore, PE exert systematic, multitarget and multichannel synergistic action due to their complex AIG [21], which hinders the analysis of the transport characteristics of PE. To evaluate the transport properties of PE more scientifically, some researchers have identified multiple components, rather than a single-component, in PE [22–24]. Nevertheless, a limited number of constituents cannot reflect the PE as a whole. However, determining all components in PE is impossible. Moreover, if diverse components in PE display completely different transport characteristics, the holistic transport characteristics of PE will be difficult to identify.

In the 1990s, bioassay systems were used to evaluate the TR of antimicrobial agents [25]. By 2005, Eguchi and his colleagues [26] had evaluated the anti-oxidative activity of carrot extract by using BL media from differentiated Caco-2 cells; this approach is more appropriate to reflect *in vivo* situations.

Based on a previous report of the activity of PhGs [9] and our results (Fig. 2), the TR of AIG in PRE can be evaluated by determining the total anti-oxidative capacity of the medium in the receiver chamber. After the transport experiments, we found that the TR of PRE was similar in both directions, and this transport was unsaturated and poorly absorbed (*’P*_app_ < 1.0 × 10^−6^ cm/s) [3], and it did not indicate efflux, suggesting that passive diffusion down a concentration gradient is the main transport mechanism of PRE (Fig. 5). Furthermore, the transport characteristics of PRE are consistent with those of AC, IS and EC, the effective components that display anti-oxidative activity in PRE (Fig. 4 and Table 4). This result was expected and indicated that the present bioassay system is appropriate and reliable for the evaluation of the transport characteristics of AIG in PRE in differentiated Caco-2 cells.

In contrast to the canonical *P*_app_ value, which is commonly used to reflect the transport of a single compound, the *’P*_app_ value obtained from the TR based on the concentration-effect curve may not only reflect the components that penetrate monolayers with an intact structure but also involve other factors related to the target activity, such as the components’ metabolites, chemically degraded products, and even some cytokines secreted from Caco-2 cells in response to stimulation that can affect the target bioactivity. Thus, the multifactor mentioned above, rather than only the transported intact components, determine the *’P*_app_ value. Therefore, *’P*_app_ may correlate more strongly with *in vivo* situations than the canonical *P*_app_ value [26]. In this study, we clarified the passively diffused and poorly absorbed nature of PRE based on its ‘*P*_app_ value, and this nature may be related to the high dosage and long clinical treatment period of CD [27]. Nevertheless, these findings will provide more systematic guidance for clinicians in the application of CD when only the transport characteristics of most AIG in CD, not only PhGs, have been identified.

However, the selected bioactivity item must be sufficiently sensitive to be detected in the medium of the receiver chamber, which presents a challenge to the establishment of a bioassay system to evaluate the transport characteristics of PE. Furthermore, the bioactivity should also depend on as many components as possible. In our study, the total anti-oxidative capacity assay was more appropriate than several other methods (S1 Fig.). But even so, our assay can only detect the TR of PRE at 120 min.

Collectively, the present study established a novel bioassay system to evaluate the intestinal permeability of PRE in differentiated Caco-2 cells. The obtained results showed that a poorly absorbed passive diffusion down a concentration gradient without efflux is the main transport mechanism of PRE (Fig. 5), which provides the pharmacokinetic basis for the clinical application of PhGs in CD. The use of a novel bioassay system to evaluate the TR and thereby calculate the *’P*_*app*_ values to assess the intestinal transport property of AIG in PEs is clearly feasible. This approach may also be suitable for other PE given appropriate bioactivity.

## Supporting Information

S1 Fig Effects of PRE on (A) superoxide anion, (B) hydroxyl radical, (C) lipid peroxidation product and (D) DPPH radical. The assay procedures were performed according to previous reports [1, 2] without using transport buffer. The IC_50_ (50% inhibition concentration) and SC_50_ (50% scavenging concentration) values were calculated based on the standard concentration-response curves.
(DOCX)
