# Supplementary material for: Evaluation of the Intestinal Transport of a Phenylethanoid Glycoside-Rich Extract from Cistanche deserticola across the Caco-2 Cell Monolayer Model
Source: PLoS One. 2015 Feb 3;10(2):e0116490. doi: 10.1371/journal.pone.0116490 (PMC4315399; doi:10.1371/journal.pone.0116490)
Supplement: S1 Fig — The assay procedures were performed according to previous reports [1, 2] without using transport buffer. The IC50 (50% inhibition concentration) and SC50 (50% scavenging concentration) values were calculated based on the standard concentration-response curves. (DOCX) [file pone.0116490.s001.docx]

**Supplemental data**

**Figure S1. Effects of PRE on (A) superoxide anion, (B) hydroxyl radical, (C) lipid peroxidation product and (D) DPPH radical.** The assay procedures were performed according to previous reports [1, 2] without using transport buffer. The IC_50_ (50% inhibition concentration) and SC_50_ (50% scavenging concentration) values were calculated based on the standard concentration-response curves.


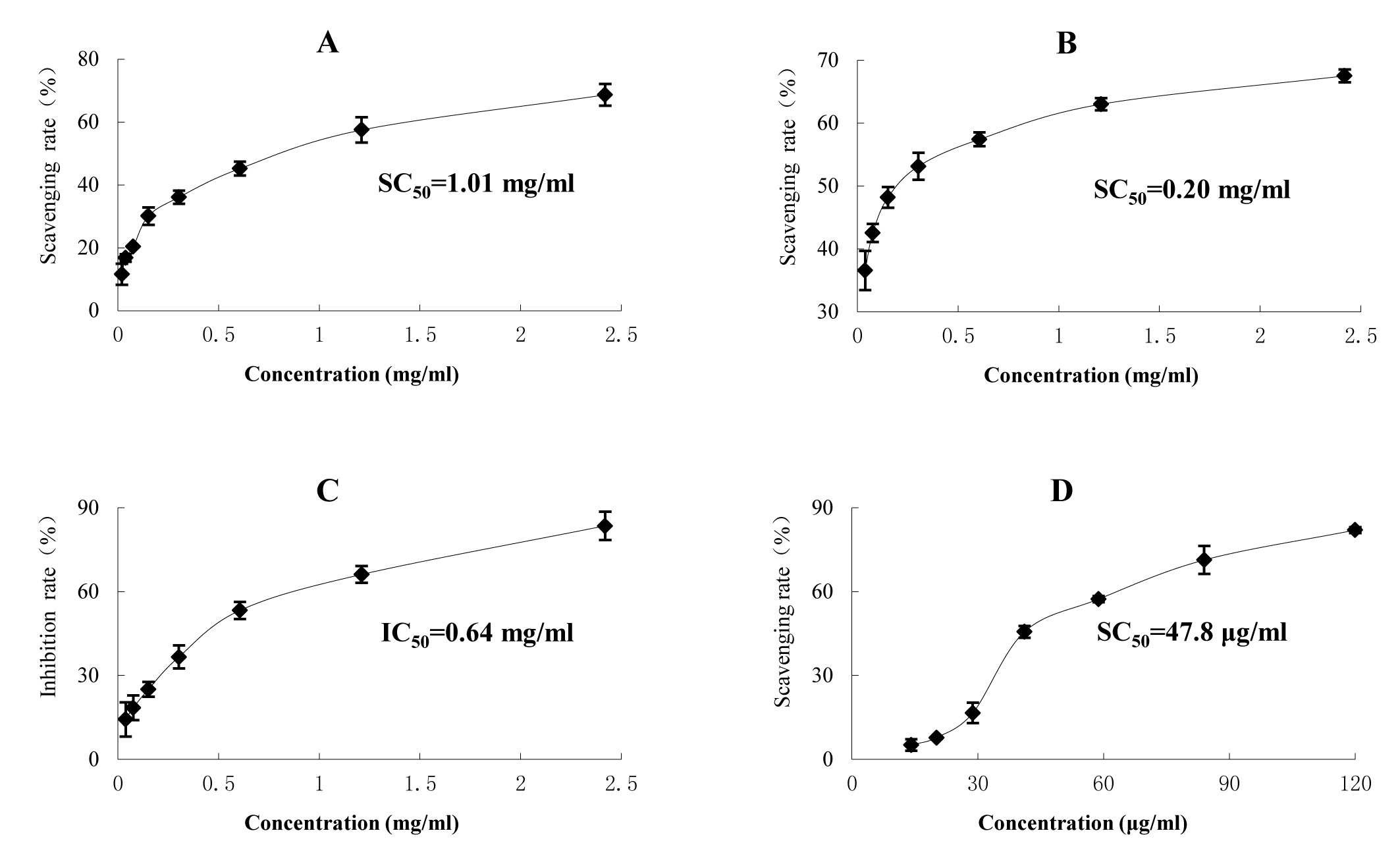


As shown in Figure S1, except for DPPH radical, the anti-oxidative effects of PRE on the other three oxidative products are all on the order of milligram per milliliter. However, a precipitate appeared as the anhydrous ethanol DPPH solution was added to the receiver chamber medium in the Caco-2 cell monolayer. Thus, the DPPH radical scavenging assay method was not suitable for our study.

**References**

1. Wang M, Xie C, Cai RL, Li XH, Luo XZ, et al. (2008) Studies on antioxidant activities of breviscapine in the cell-free system. Am J Chin Med 36: 1199-1207.
2. Halliwell B, Gutteridge JM, Aruoma OI (1987) The deoxyribose method: a simple "test-tube" assay for determination of rate constants for reactions of hydroxyl radicals. Anal Biochem 165: 215-219.
